# Regular use of aspirin is associated with a lower cardiovascular risk in prostate cancer patients receiving gonadotropin-releasing hormone therapy

**DOI:** 10.3389/fonc.2022.952370

**Published:** 2022-09-12

**Authors:** Wei-Ting Chang, Chon-Seng Hong, Kun-Lin Hsieh, Yi-Chen Chen, Chung−Han Ho, Jhih-Yuan Shih, Wei-Chih Kan, Zhih-Cherng Chen, You-Cheng Lin

**Affiliations:** ^1^ Division of Cardiology, Department of Internal Medicine, Chi Mei Medical Center, Tainan, Taiwan; ^2^ Department of Biotechnology, Southern Taiwan University of Science and Technology, Tainan, Taiwan; ^3^ Institute of Clinical Medicine, College of Medicine, National Cheng Kung University, Tainan, Taiwan; ^4^ Department of Health and Nutrition, Chia Nan University of Pharmacy and Science, Tainan, Taiwan; ^5^ Division of Urology, Department of Surgery, Chi-Mei Medical Center, Tainan, Taiwan; ^6^ Department of Environmental and Occupational Health, College of Medicine, National Cheng Kung University, Tainan, Taiwan; ^7^ Department of Medical Research , Chi-Mei Medical Center, Tainan, Taiwan; ^8^ Department of Health and Nutrition , Chia Nan University of Pharmacy and Science, Tainan, Taiwan; ^9^ Division of Nephrology, Department of Internal medicine, Chi Mei Medical Center, Tainan, Taiwan; ^10^ Department of Biological Science and Technology, Chung Hwa University of Medical Technology, Tainan, Taiwan; ^11^ Division of Plastic Surgery, Department of Surgery, Chi-Mei Medical Center, Tainan, Taiwan

**Keywords:** prostate cancer, GnRH therapy, aspirin, cardiotoxicity, MACCEs

## Abstract

Gonadotropin-releasing hormone (GnRH) therapy has been known to increase risks of major adverse cardiovascular and cerebrovascular events (MACCEs). Herein, we aim to estimate whether regular use of aspirin attenuates risks of MACCEs in prostate cancer patients receiving GnRHs. Using Taiwanese National Health Insurance Research Database (NHIRD), we identified 7719 patients diagnosed with prostate cancer who were either aspirin-naïve, received irregular or regular aspirin from 2008 to 2015. Through a multivariable logistic regression model, we investigated the impact of aspirin on MACCEs. Compared with nonusers and irregular users, most patients receiving regular aspirin were older and had more comorbidities. The crude incidence of one-year MACCEs was lowest in aspirin nonusers but highest in irregular users of aspirin compared with regular users of aspirin (2.65% vs. 4.41% vs. 2.85%, p=0.0099). After adjusting for age, cancer stage and comorbidities, irregular aspirin users had a higher risk of one-year MACCEs (adjusted OR: 1.33; 95% CI: 0.93-1.90, p=0.1139) than aspirin nonusers, but conversely, there was a trend of reducing the risk of MACCEs among those who received regular aspirin (adjusted OR: 0.79; 95% CI: 0.44-1.42, p=0.4256). In the subgroup analysis, there were age- and cancer stage-independent higher risks of MACCEs in patients who took aspirin irregularly compared to those in patients who did not take aspirin. The risks were attenuated in patients receiving regular aspirin. Collectively, regular use of aspirin presented a trend of reducing risks of MACCEs in prostate cancer patients receiving GnRHs. However, irregular use of aspirin diminished the benefits.

## Introduction

With a high prevalence, prostate cancer affects approximately one man in eight during his lifetime (1, 2). Notably, at the average age of 66 at diagnosis, most prostate cancer patients were at old ages and had comorbidities (2). Medical castration, including gonadotropin-releasing hormone (GnRH) therapy, has significantly improved cancer-free survival (3, 4). Nevertheless, since the first observational study in a Surveillance, Epidemiology, and End Results-Medicare database, numerous studies, including randomized controlled trials, have identified an increased risk of incident coronary heart disease and myocardial infarction in association with GnRH therapy (5–7). To date, the cornerstone of management of GnRH therapy-associated cardiovascular complications relies on prevention (6, 7). Before initiating GnRH therapy, a multidisciplinary discussion with the patient, especially those with multiple cardiovascular risk factors, about the risks and benefits of GnRH therapy should be carried out (6). Additionally, increasing the awareness of patients of the signs of cardiovascular disease and screening for undiagnosed risk factors are essential (6, 8). Notably, evidence suggests that, in patients diagnosed with high-risk prostate cancer, aspirin may be associated with lower prostate cancer-specific mortality (9, 10). Although this has yet to be validated in other populations, it is possible that select patients may benefit from aspirin for primary and secondary prevention of cardiovascular diseases and possibly for reduced cancer-related mortality.

Aspirin, through its anti-inflammatory effect, can inhibit tumor cell growth and the development of cardiovascular diseases (9, 10). By suppressing cyclooxygenase 1/2, some preclinical studies have found some possible antitumor mechanisms of aspirin in prostate cancer (11, 12). Downer et al. suggested that regular aspirin lowers the risk of lethal prostate cancer (13). Additionally, evidence suggests that, in patients diagnosed with high-risk prostate cancer, aspirin may be associated with lower prostate cancer-specific mortality (9, 10). Nevertheless, it remains uncertain whether prostate cancer patients may benefit from aspirin for primary and secondary prevention of cardiovascular diseases. Hereby, using a nationwide database, we aimed to study whether regular aspirin use is associated with a risk reduction of major adverse cardiovascular and cerebrovascular events (MACCEs) in prostate cancer patients receiving GnRH therapy.

## Method

### Data source

This study was a real-world database study using Taiwan’s National Health Insurance Research Database (NHIRD) and the Taiwan Cancer Registry (TCR), which are released by the Health and Welfare Data Science Center (HWDC). The NHIRD includes age, sex, medications, procedures and all medical diagnoses from Taiwan’s single-payer National Health Insurance program. The diagnosis of diseases and procedures in the NHIRD was based on the International Classification of Diseases (ICD) codes, so high reliability could be confirmed (14). In addition, the TCR is a high-quality registry database that includes almost 97% of cancer patients and has been established by Taiwan’s Ministry of Health and Welfare since 1979 (15, 16). Information on age at cancer diagnosis, sex, cancer stages, cancer sites, tumor histology, and treatment types are all present in the TCR. The institutional review committee of Chi Mei Medical Center approved this study (IRB: 11005-E03). Encrypted personal identification was used to prevent the possibility of an ethical violation according to the regulations of the Bureau of National Health Insurance (BNHI) in Taiwan, so informed consent was not required.

### Definitions of studied subjects

**Figure 1:**
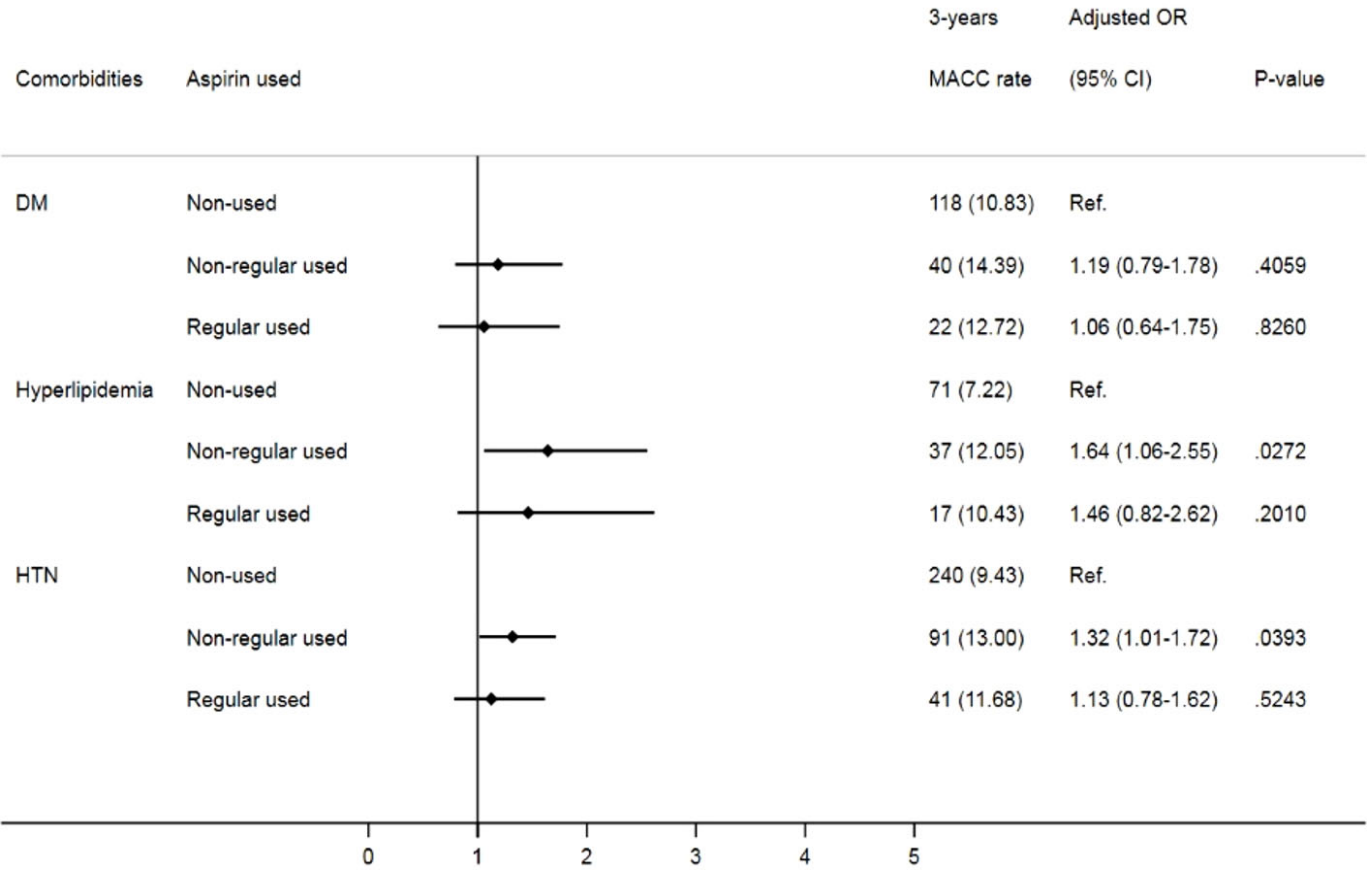
The flowchart of study design and patient selection.

Patients newly diagnosed with prostate cancer and receiving GnRH therapies from 2008 to 2015 were identified from the TCR-long form, and the detailed information is listed in **Figure 1**. To correspond to the aim of the completed dataset, the initial exclusion criteria for this study were patients with a history of MACCEs before cancer, incomplete medical records of cancer stage, and patients aged less than 18 years. Moreover, we defined patients who only received medical castration, including GnRH agonists/antagonists, within 2 years after the date of diagnosis of cancer as the target patients. Thus, the index date of this study was the date of prostate cancer patients receiving GnRH therapy. Patients given surgical castration or receiving both GnRH and surgical therapy, patients who received treatment before cancer diagnosis, patients receiving GnRH therapy two years after a cancer diagnosis, and MACCEs before medical castration were all excluded. Finally, patients with a history of aspirin use were assessed within one year before the diagnosis of cancer. All patients were divided into three groups: (1) patients with aspirin prescription for more than 10 months in one year were regular aspirin users, (2) otherwise patients were defined as irregular users of aspirin. Also, (3) patients without aspirin use at least one year before the diagnosis of cancer were aspirin-naïve. The dosage of aspirin was 100mg per day.

Information regarding age, clinical stage of cancer, comorbidities, and concomitant medication history within the previous 12 months among the study subjects was also considered a potential confounding factor. Age was classified into the following groups: <60, 60-69, and ≧70 years old. The clinical stage of cancer included early stages (I/II) and advanced stages (III/VI). The diagnosis codes of comorbidities and the Anatomical Therapeutic Chemical (ATC) codes of drugs are shown in **Supplementary Table 1**.

### Study endpoint

The primary outcome was a composite endpoint of MACCEs, including new-onset acute myocardial infarction (AMI), heart failure (HF), and ischemic stroke (including transient ischemic attack [TIA]), after the index date during the study period. All patients were followed-up from the index date to death, were lost to follow-up, or were followed the whole three years. The sensitivity analysis approach was also used to verify MACCE risk. Both one-year and three-year risks of MACCEs were estimated to validate the association between MACCEs and three groups of aspirin use. Because ICD-9-CM was replaced by ICD-10-CM in 2016, both ICD-9-CM and ICD-10-CM codes were used to identify endpoints in the primary outcome during the follow-up period (**Supplementary Table 1**).

### Statistical analysis

The baseline information of patients is presented as numbers with percentages, and the differences in proportions were evaluated using Pearson’s chi-square test or Fisher’s exact test. A multivariable logistic regression model was used to evaluate the risk of MACCEs among the three aspirin use groups. Patient age, clinical stage of cancer, comorbidities, and the use of drugs were included as covariates in the model to estimate the adjusted odds ratio (OR) with the 95% confidence interval (95% CI). Subgroup analyses of different age groups and cancer stages were also performed. A forest plot was used to present the risk of MACCEs between the three aspirin use groups among patients with diabetes, hyperlipidemia or hypertension. Statistical significance was considered a p-value less than 0.05. SAS 9.4 for Windows (SAS Institute Inc., Cary, NC, USA) was used for all data analyses.

## Results

### Demographic information of prostate cancer patients

From 2008 to 2015, we identified 7719 patients diagnosed with prostate cancer with information on aspirin use. Among them, 6346 patients were aspirin-naïve, 952 patients were irregular aspirin users and 421 patients were regular aspirin users (**Table 1**). Compared with aspirin nonusers and irregular aspirin users, most patients who received regular aspirin were older and were diagnosed at an earlier cancer stage. There were more cardiovascular comorbidities, including diabetes, hypertension, hyperlipidemia, peripheral artery disease, valvular heart diseases, atrial fibrillation, and chronic kidney disease, among patients with regular aspirin use compared to that among aspirin nonusers or irregular aspirin users. Additionally, more regular aspirin users were prescribed ACEIs/ARBs and statins. Although the crude incidence of all-cause mortality was not significantly different among groups, interestingly, the one-year crude incidence of MACCEs was lowest in aspirin nonusers but highest in irregular aspirin users compared with those in regular aspirin users (2.65% vs. 4.41% vs. 2.85%, p=0.0099). The crude incidence was persistently lower in aspirin nonusers compared to those in the other two groups at the three-year follow-up.

**Table 1:** Demographic information of prostate cancer patient in groups of non-Aspirin use, non-regular Aspirin use and regular Aspirin use.

	Non-Aspirin use N = 6,346	Non-regularAspirin use N = 952	Regular Aspirin use N = 421	P-value
Age groups				
<60	645 (10.16)	38 (3.99)	14 (3.33)	<.0001
60-69	1849 (29.14)	238 (25.00)	92 (21.85)	
≧70	3852 (60.70)	676 (71.01)	315 (74.82)	
Clinical stage				
I, II	1764 (27.80)	296 (31.09)	136 (32.30)	0.0216
III, IV	4582 (72.20)	656 (68.91)	285 (67.70)	
Mortality	2328 (36.68)	371 (38.97)	151 (35.87)	0.3552
MACCEs				
1-years	168 (2.65)	42 (4.41)	12 (2.85)	0.0099
3-years	483 (7.61)	105 (11.03)	47 (11.16)	0.0001
Comorbidities				
PAD	54 (0.85)	19 (2.00)	10 (2.38)	0.0002
DM	1090 (17.18)	278 (29.20)	173 (41.09)	<.0001
Hyperlipidemia	983 (15.49)	307 (32.25)	163 (38.72)	<.0001
Valvular heart disease	72 (1.13)	32 (3.36)	15 (3.56)	<.0001
Asthma	200 (3.15)	33 (3.47)	14 (3.33)	0.8661
Atrial fibrillation	51 (0.80)	36 (3.78)	24 (5.70)	<.0001
CKD	465 (7.33)	101 (10.61)	40 (9.50)	0.0009
HTN	2546 (40.12)	700 (73.53)	351 (83.37)	<.0001
COPD	352 (5.55)	76 (7.98)	34 (8.08)	0.0023
Drug used				
ACEI	345 (5.44)	119 (12.50)	73 (17.34)	<.0001
Anti-coagulants: Warfarin	54 (0.85)	15 (1.58)	3 (0.71)	0.0846
ARB	1157 (18.23)	362 (38.03)	221 (52.49)	<.0001
Statin	607 (9.57)	215 (22.58)	133 (31.59)	<.0001
Cyproterone, bicalutamide and diethylstilbestrol	189 (2.98)	27 (2.84)	13 (3.09)	0.9604

P-value was from Pearson’s chi-square test.

MACCEs, major adverse cardiovascular and cerebrovascular events; PAD, peripheral arterial disease; DM, diabetes mellitus; HTN, hypertension; CKD,chronic kidney disease; COPD,chronic obstructive pulmonary disease; ACEI, Angiotensin Converting Enzyme Inhibitors; ARB, Angiotensin II receptor blockers.

### The incidence of mortality and MACCEs between aspirin users and nonusers

After adjusting for age, comorbidities, cardiovascular medications and androgen receptor antagonists, in terms of one-year MACCEs, compared with aspirin nonusers, irregular aspirin users had a relatively higher risk of MACCEs (adjusted OR: 1.33; 95% CI: 0.93-1.90, p=0.1139), while notably, there was a trend of reducing the risk of MACCEs among those who received aspirin regularly (adjusted OR: 0.79; 95% CI: 0.44-1.42, p=0.4256) (**Table 2**). Additionally, other risk factors, including old age (≥70 years old) (adjusted OR: 3.31; 95% CI: 1.51-7.25, p<0.0028); advanced cancer stage (III/VI) (adjusted OR: 1.44; 95% CI: 1.05-1.97, p<0.0256); and comorbidities, including DM (adjusted OR: 1.65; 95% CI: 1.21-2.25, p=0.0015), HTN (adjusted OR: 1.44; 95% CI: 1.04-2.00, p=0.0279) and CKD (adjusted OR: 1.57; 95% CI: 1.07-2.23, p=0.0223), were associated with the risk of MACCEs at the one-year follow-up. Given the instantaneous risk of mortality among patients with prostate cancer, we adjusted the ORs with mortality as a competing event. As MACCEs are a leading cause of death, the results also indicated a relatively higher risk of one-year MACCEs (adjusted OR: 1.30; 95% CI: 0.91-1.85, p=0.1487) in irregular users of aspirin compared to that in aspirin nonusers, while the risk was slightly reduced among those who received regular aspirin (adjusted OR: 0.77; 95% CI: 0.43-1.39, p=0.3813).

**Table 2:** Risks of 1-year and 3-years major adverse cardiovascular and cerebrovascular events (MACCEs) in prostate cancer patients having different treatment or comorbidities.

	1- year MACCEs				3-years MACCEs			
	Adjusted OR(95% C.I.)	P-value	Adjusted OR^a^(95% C.I.)	P-value	Adjusted OR(95% C.I.)	P-value	Adjusted OR^a^(95% C.I.)	P-value

Non-used	Ref.		Ref.		Ref.		Ref.	
Non-regular aspirin used	1.33 (0.93-1.90)	0.1139	1.30 (0.91-1.85)	0.1487	1.18 (0.94-1.49)	0.1608	1.14 (0.90-1.43)	0.2824
Regular aspirin used	0.79 (0.44-1.42)	0.4256	0.77 (0.43-1.39)	0.3813	1.08 (0.78-1.51)	0.6321	1.04 (0.75-1.45)	0.7988
Age groups								
<60	Ref.		Ref.		Ref.		Ref.	
60-69	1.79 (0.78-4.09)	0.1692	1.79 (0.78-4.08)	0.1694	1.29 (0.83-2.01)	0.2532	1.29 (0.83-2.01)	0.2521
≧70	3.31 (1.51-7.25)	0.0028	3.35 (1.53-7.34)	0.0025	2.54 (1.69-3.83)	<.0001	2.61 (1.74-3.94)	<.0001
Clinical stage								
I, II	Ref.		Ref.		Ref.		Ref.	
III. IV	1.44 (1.05-1.97)	0.0256	1.46 (1.07-2.01)	0.0186	1.20 (0.99-1.44)	0.0627	1.18 (0.98-1.42)	0.0785
Comorbidities								
PAD	1.38 (0.52-3.65)	0.5158	1.36 (0.51-3.59)	0.5393	1.69 (0.92-3.10)	0.0917	1.63 (0.89-2.99)	0.1163
DM	1.65 (1.21-2.26)	0.0015	1.62 (1.18-2.20)	0.0024	1.51 (1.24-1.85)	<.0001	1.51 (1.24-1.84)	<.0001
Hyperlipidemia	0.72 (0.48-1.08)	0.1089	0.71 (0.48-1.07)	0.0995	0.82 (0.64-1.05)	0.1176	0.82 (0.64-1.05)	0.1185
Valve	1.27 (0.54-3.02)	0.5871	1.24 (0.52-2.95)	0.6275	2.05 (1.27-3.31)	0.0034	2.10 (1.31-3.37)	0.0021
Asthma	1.27 (0.65-2.45)	0.4857	1.25 (0.65-2.42)	0.5058	1.19 (0.78-1.81)	0.4296	1.27 (0.84-1.91)	0.2612
AF	0.91 (0.34-2.44)	0.8469	0.90 (0.33-2.42)	0.8313	1.21 (0.68-2.17)	0.5155	1.15 (0.64-2.06)	0.6385
CKD	1.57 (1.07-2.32)	0.0223	1.60 (1.09-2.35)	0.0161	1.34 (1.03-1.74)	0.0284	1.35 (1.04-1.74)	0.0248
HTN	1.44 (1.04-2.00)	0.0279	1.48 (1.07-2.05)	0.0169	1.27 (1.04-1.55)	0.0207	1.29 (1.06-1.58)	0.0124
COPD	1.36 (0.85-2.16)	0.1989	1.41 (0.89-2.22)	0.1432	1.24 (0.91-1.67)	0.1708	1.29 (0.96-1.73)	0.0949

^a^MACCE is the leading cause of death.

Abbreviations as listed in **Table 1**.

### Subgroup analysis focusing on age and cancer stage among aspirin nonusers and regular and irregular users

Given that prostate cancer patients who were aspirin nonusers had fewer cardiovascular risk factors, to prevent bias, we thereafter defined the risks of aspirin nonusers as the reference and focused on the comparison between those with regular or irregular aspirin use (**Table 3**). As categorized by age, among patients at relatively younger ages from 60 to 69 years old, the risk of one-year MACCEs was higher in patients who took aspirin irregularly compared to patients who were aspirin nonusers (adjusted OR: 2.65; CI: 1.23-5.71, p=0.0127). Notably, among patients who received aspirin regularly, the risks decreased and became insignificant (adjusted OR: 2.28; CI: 0,68-7.64, p=0.1837). In contrast, for patients aged above 70 years, the risks of one-year MACCEs were persistently higher in both irregular aspirin users (OR: 1.41; 95% CI: 0.96-2.09, p= 0.0820) and irregular aspirin users (OR: 0.81; 95% CI: 0.41-1.61, p= 0.5459) compared to that in aspirin nonusers. To avoid competing risks of death, we adjusted the OR with mortality and found a persistently higher risk of one-year MACCEs among patients who took aspirin irregularly (adjusted OR: 2.62; 95% CI: 1.15-5.96, p=0.0221 for the 60- to 69-year-old age group) compared to those free from aspirin use, while the risk was insignificant among those who received aspirin regularly (adjusted OR: 1.99; 95% CI: 0.56-7.13, p=0.2905).

**Table 3:** Risks of 1-year and 3-years mortality and major adverse cardiovascular and cerebrovascular events (MACCEs) of Aspirin use in prostate cancer patient with different ages or cancer stages.

	1- year						3-years				
		MACC						MACC			
	Mortality (%)	Crude OR(95% C.I.)	P-value	Adjusted OR^b^(95% C.I.)	P-value		Mortality(%)	Crude OR(95% C.I.)	P-value	Adjusted OR^b^(95% C.I.)	P-value
**Age groups**										
60-69										
No used	60(3.24)	Ref.		Ref.			308(16.66)	Ref.		Ref.	
Non-regular aspirin used	10(4.20)	2.65 (1.23-5.71)	0.0127	2.62 (1.15-5.96)	0.0221		35(14.71)	1.93 (1.16-3.20)	0.0113	1.78(1.03-3.05)	0.0383
Regular aspirin used	5(5.43)	2.28 (0.68-7.64)	0.1837	1.99(0.56-7.13)	0.2905		8(8.70)	1.47 (0.62-3.45)	0.3812	1.30(0.53-3.20)	0.5656
70≧										
No used	306(7.94)	Ref.		Ref.			1013(26.30)	Ref.		Ref.	
Non-regular aspirin used	56(8.28)	1.41 (0.96-2.09)	0.0820	1.20(0.80-1.80)	0.3847		194(28.70)	1.32 (1.02-1.69)	0.0329	1.11(0.86-1.45)	0.4220
Regular aspirin used	21(6.67)	0.81 (0.41-1.61)	0.5459	0.63(0.31-1.27)	0.1943		82(26.03)	1.39 (0.98-1.96)	0.0626	1.07(0.74-1.54)	0.7176
**Clinical stage**										
I, II										
No used	35(1.98)	Ref.		Ref.			155(8.79)	Ref.		Ref.	
Non-regular aspirin used	4(1.35)	0.89 (0.38-2.12)	0.7956	0.73(0.30-1.77)	0.4822		24(8.11)	1.32 (0.85-2.06)	0.2203	1.14(0.72-1.82)	0.5834
Regular aspirin used	<3	0.97 (0.30-3.18)	0.9628	0.69(0.20-2.37)	0.5594		12(8.82)	1.57 (0.88-2.82)	0.1284	1.12(0.60-2.09)	0.7151
III. IV										
No used	351(7.66)	Ref.		Ref.			1293(28.22)	Ref.		Ref.	
Non-regular aspirin used	62(9.45)	2.02 (1.38-2.95)	0.0003	1.53(1.03-2.29)	0.0365		212(32.32)	1.59 (1.23-2.06)	0.0004	1.19(0.91-1.57)	0.2026
Regular aspirin used	25(8.77)	1.14 (0.57-2.26)	0.7184	0.78(0.39-1.60)	0.5024		79(27.72)	1.52 (1.04-2.22)	0.0296	1.06(0.71-1.57)	0.7910

^b^Adjusted for risk factor described in **Table 1**.

In terms of cancer stages, the use of aspirin did not show a significant impact on the risks of MACCEs in patients at a relatively earlier cancer stage (stage I/II). In contrast, among patients at an advanced cancer stage (stage III/VI), irregular aspirin use presented higher risks of MACCEs than those of aspirin nonusers at the one-year follow-up (OR: 2.02, 95% CI: 1.38-2.95, p= 0.0003), while the risk was slightly reduced among patients with regular aspirin use (OR: 1.14, 95% CI: 0.57-2.26, p= 0.7184). Likewise, after adjusting for competing risks of mortality, the risk of one-year MACCEs remained higher in patients with irregular aspirin use (adjusted OR: 1.53; 95% CI: 1.03-2.29, p=0.03865) than in those free from aspirin use, while the risk was insignificant among those who received aspirin regularly (adjusted OR: 0.78; 95% CI: 0.39-1.60, p=0.5024).

### Subgroup analysis focusing on cardiovascular risk factors among aspirin nonusers and regular and irregular users

**Figure 2:**
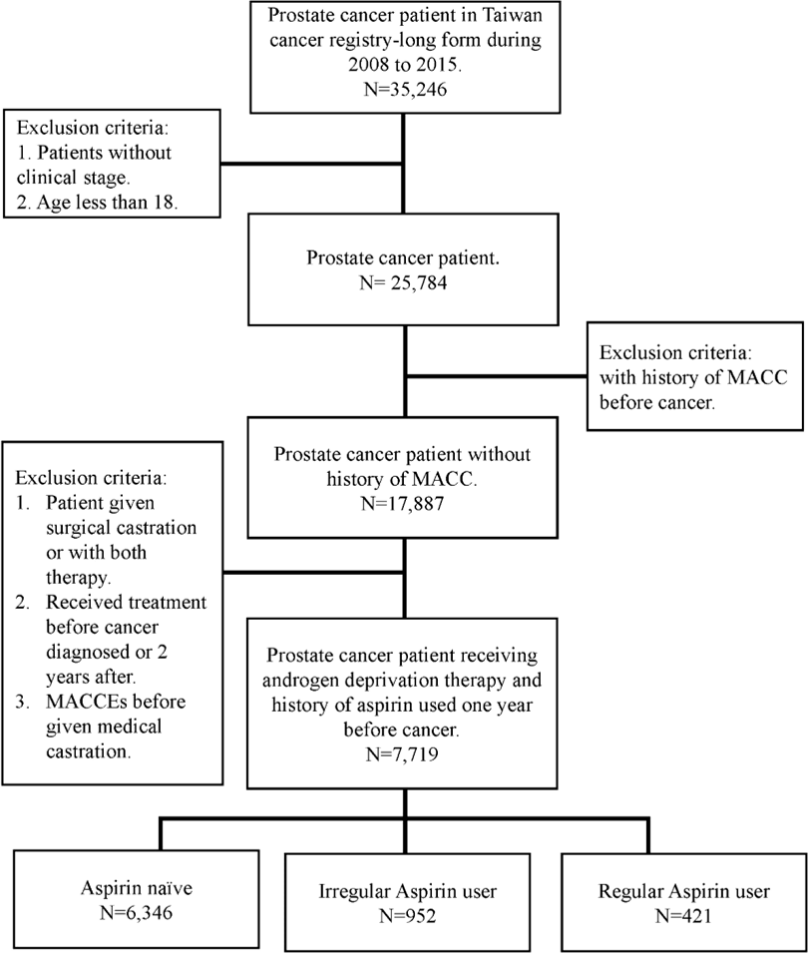
Risks of one-year MACCEs in sub-group analysis of different comorbidities. (DM, diabetes mellitus; HTN, hypertension).

**Figure 3:**
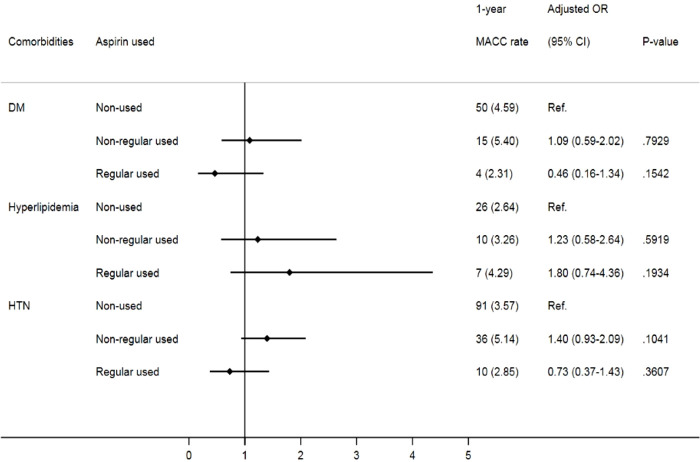
Risks of three-year MACCEs in sub-group analysis of different comorbidities. (DM, diabetes mellitus; HTN, hypertension).

Given that comorbidities, including diabetes, hypertension and hyperlipidemia, also contribute to MACCEs, we further analyzed the impact of aspirin use on prostate cancer patients with different cardiovascular risk factors. Despite no significant difference in MACCEs in the one-year follow-up **(** **Figure 2** **)**, when focusing on prostate cancer patients with underlying hypertension, we found that irregular users of aspirin had a higher risk of three-year MACCEs (OR: 1.32, 95% CI: 1.01-1.72, p= 0.0393) than those who did not use aspirin. Interestingly, the increasing risk was insignificant in patients receiving aspirin regularly (OR: 1.13, 95% CI: 0.78-1.62, p= 0.5243). Likewise, among patients with coexisting hyperlipidemia, patients receiving aspirin irregularly also showed a higher risk of three-year MACCEs (OR: 1.64, 95% CI: 1.06-2.55, p= 0.0272) than those who did not use aspirin, while the risk was diminished in those with regular aspirin use (OR: 1.46, 95% CI: 0.82-2.62, p=0.2010) **(** **Figure 3** **)**. In contrast, among patients with diabetes, the risk of MACCEs was not significantly different among the groups.

## Discussion

In this nationwide cohort focusing on prostate cancer patients, we observed that, despite a higher risk of MACCEs among irregular aspirin users compared with those free from aspirin use, after adjusting for age, cancer stage and comorbidities, there was a trend of reducing the risk of MACCEs among those who received aspirin regularly. Notably, the benefit of regular aspirin use on cardiovascular risk reduction seems to be age- and cancer stage-independent. Special attention should be given to patients with underlying cardiovascular risk factors, including concomitant hypertension or hyperlipidemia, while aspirin use may be more promising in reducing the risks of MACCEs. Although Bhatia et al. raised the concept of ABCDE steps for heart and vascular wellness following a prostate cancer diagnosis, there is a missing piece in regard to evidence supporting the use of aspirin for the prevention of cardiovascular diseases in prostate cancer patients, especially those receiving GnRH therapy (8). Our findings, for the first time, indicated that regular use of aspirin may mitigate the risks of MACCEs in prostate cancer patients who underwent GnRH therapy.

Systemic review and meta-analysis studies indicated that, through achieving castration, GnRH therapy is efficient in prostate cancer control (3, 5, 17). However, as testosterone declines, adverse effects, including metabolic disorders and MACCEs, develop (4, 7). A meta-analysis study showed that low endogenous testosterone levels are associated with cardiovascular mortality (18). The Surveillance, Epidemiology and End Results (SEER)-Medicare database reported that prostate cancer itself, prostate cancer treatments, and selection mechanisms all contribute to an increased risk of thromboembolic disease (19). In terms of pathophysiology, in a murine model of orchiectomized LDLR^−/−^ mice, mice fed high-fat diets developed larger atherosclerotic plaques compared to those in sham mice (20). In contrast, testosterone supplementation in the orchiectomy model significantly reduced atherosclerotic lesion size (20). Additionally, in macrophages, testosterone augmented cholesterol efflux in a dose-dependent manner, suggesting a possible mechanism by which testosterone can reduce atherosclerotic lesions (21).

Chronic inflammation is a major contributor to human cancers and exacerbates metabolic and cardiovascular disorders (22). Also, in a review article Camilli et al. highlighted the importance of the crosstalk between cancer and platelet which promotes cancer-associated inflammation, proliferation and immune (23). Aspirin is a commonly used medication with anti-platelet activity ad anti-inflammatory (9, 12, 13). Beyond its widely used application in reducing the risks of ischemic vascular diseases, there is also evidence from epidemiological and interventional studies to suggest that regular aspirin use may reduce the risk of prostate cancer development and progression (9, 12, 13). A systematic review and meta-analyses of 118 observational studies of aspirin indicated that an around 20% reduction in mortality in cancer patients receiving aspirin (24). By suppressing cyclooxygenase-2 enzyme-mediated inflammation, aspirin can modify prostate cancer biology and disease characteristics (12). Nevertheless, the evidence regarding the impact of aspirin on the risks of cancers remains controversial (9, 10, 12, 13). In a recent analysis of a large clinical database, postdiagnosis aspirin use was associated with 57% lower prostate cancer-specific mortality (25). Through large cohort studies, Cao et al. also found that long-term aspirin use was associated with a modest but significantly reduced risk of overall cancer (26). Likewise, using the Cancer Prevention Study-II Nutrition Cohort, Jacobs et al. reported that compared with no aspirin use and neither prediagnosis nor postdiagnosis, daily aspirin use was significantly associated with the survival of prostate cancer patients (27). In a systematic review and meta-analysis, Liu et al. also demonstrated that there was no significant association between aspirin use and the risk of prostate cancer-associated mortality (28). Given the increasing risks of MACCEs in prostate cancer patients receiving GnRH therapy, the use of aspirin may further benefit cardiovascular outcomes beyond its effect on cancer survival (12, 26, 28). Therefore, there has been a precent of aspirin already used in patients with prostate cancer in order to reduce the risks associated with treating the disease (9, 25, 27, 29). However, although Li et al. showed that aspirin was associated with lower venous thrombosis and overall in-hospital mortality in older patients with cancer, there is a lack of evidence supporting the effect of aspirin use on cardiovascular risks in patients receiving GnRH therapy (29). Our findings, at least in part, provide missing evidence for the use of aspirin in reducing the risks of MACCEs in prostate cancer patients.

In a recently published cross-sectional analysis among 90,494 US veterans with prostate cancer, 54.1% had uncontrolled risk factors, but among them, 29.6% were not receiving risk-reducing medication (30). This study emphasized gaps in care and the need for interventions in this population but lacked investigations focusing on interventions for cardiovascular drugs, such as aspirin (30). To provide missing evidence, our findings highlight the importance of regular use of aspirin in reducing cardiovascular risks in prostate cancer patients receiving GnRH therapy. Notably, compared with aspirin-naïve patients, we found that those with irregular aspirin use had a higher risk of MACCEs. However, it is arbitrary jumping to the conclusion that aspirin fails to reduce cardiovascular risks given that patients who were prescribed aspirin may have increasing cardiovascular risks at baseline. In contrast, regular use of aspirin attenuated the risks of MACCEs compared with its irregular use. This underlines the issue of underassessed and undertreated cardiovascular risk factors in patients with prostate cancer, which seriously requires more attention. Especially in patients with old age, advanced cancer stage or comorbidities (such as hypertension and hyperlipidemia), the importance of regular use of aspirin is even more promising in improving cardiovascular outcomes. Although it may be premature to recommend aspirin for all patients receiving GnRH therapy, cautioning patients that irregular use may be detrimental is worth considering. Most importantly, given the ADD-aspirin trial underway, further randomized trials will benefit in illustrating the necessity of aspirin use among men diagnosed with prostate cancer.

There are some limitations to this study. First, given the retrospective study design, patients were not randomized to aspirin. Even though the study was designed under a matching method to reduce the difference between groups, there is still bias due to background cardiovascular risk factors of patients, especially those prescribed aspirin. Second, owing to database-type studies, we could not obtain full detailed information, including the integrated lifestyle-related factors of patients and the severity of MACCEs. Third, in the subgroup analysis, among patients with DM, which is also known as coronary artery disease risk equivalent, aspirin use failed to represent a beneficial effect of risk reductions. Notably, there was a significantly higher ratio of regular aspirin use than aspirin-naïve and irregular aspirin use in patients with DM. As speculated, in this population with high adherence to aspirin, the difference between irregular and regular aspirin use may be insignificant, but the importance of regular aspirin use in high-risk populations is not ignorable.

This study indicated that regular use of aspirin was with a trend of reducing risks of MACCEs in prostate cancer patients who receive GnRH therapy. However, irregular aspirin use diminished the benefits. To date, even though regular aspirin use is not yet recommended in practice guidelines, the high burden of cardiovascular diseases among prostate cancer patients requires special attention. Therefore, the major value of this study is by setting out the immediate clinical implications of aspirin use in patients with prostate cancer. Also, it is necessary for a randomized trial to illustrate the necessity of aspirin use among patients with prostate cancer. In summary, seems a valuable contribution to the literature and perhaps highlights the importance of the cardio-oncologist as cancer patients may also have elevated risks of adverse cardiovascular outcomes.

## Data availability statement

The data analyzed in this study is subject to the following licenses/restrictions: The data is restrictive only to the NHIRD. Requests to access these datasets should be directed to cmcvecho3@gmail.com.

## Ethics statement

The studies involving human participants were reviewed and approved by IRB: 11005-E03. Written informed consent for participation was not required for this study in accordance with the national legislation and the institutional requirements.

## Author contributions

Study concept and design, W-TC. Acquisition of data, Y-CC and C-HH. Analysis and interpretation of data, Y-CC and C-HH. Drafting of the manuscript, J-YS, C-SH, K-LH, and W-TC. Critical revision of the manuscript for important intellectual content, J-YS, C-SH, K-LH, Y-CC, C-HH, J-YS, Z-CC, and W-TC. Statistical analysis, Y-CC and C-HH. Obtaining funding, C-SH and W-TC. Supervision, W-TC. All authors contributed to the article and approved the submitted version.

## Funding

This work was granted by Ministry of Science and Technology (MOST 109-2326-B-384 -001 -MY3). This study was also supported by Chi-Mei Medical Center.

## Conflict of interest

The authors declare that the research was conducted in the absence of any commercial or financial relationships that could be construed as a potential conflict of interest.

## Publisher’s note

All claims expressed in this article are solely those of the authors and do not necessarily represent those of their affiliated organizations, or those of the publisher, the editors and the reviewers. Any product that may be evaluated in this article, or claim that may be made by its manufacturer, is not guaranteed or endorsed by the publisher.
