# Supplementary material for: Regular use of aspirin is associated with a lower cardiovascular risk in prostate cancer patients receiving gonadotropin-releasing hormone therapy
Source: Front Oncol. 2022 Sep 12;12:952370. doi: 10.3389/fonc.2022.952370 (PMC9511956; doi:10.3389/fonc.2022.952370)
Supplement: Supplementary file 1 [file Table_1.docx]

**Supplementary Table 1**

| **Comorbidities** | **ICD-9 Codes** | **ICD-10 Codes** |
| --- | --- | --- |
| Peripheral artery disease (PAD) | 443., 440, 443, 444, 447.8, 447.9 | I73.9,I70.2-I70.9, I71, I74.2, I74.3, I74.4, I74.5, I77.89, I77.9 |
| Diabetes mellitus (DM) | 250.xx | E08.xx, E09.xx, E11.xx, E13.xx |
| Hyperlipidemia | 272.xx | E78.xx |
| Valvular heart disease | 394.x-397.x,424.0,424.1,424.2,424.3 | I05.x, I06.x, I07.x, I08.x, I09.x, I34.xx-I37.xx |
| Asthma | 493.xx | J45.xx |
| Atrial fibrillation (AF) | 427.31,427.32 | I48.xx |
| Chronic kidney disease (CKD) | 580.xx -589.xx, 403, 404, 585, V45.1, V56 | I12.x, I13.x, N02.x, N03.x, N04.x, N05.x, N06.x, N07.x, N08, N11.x, N14.x, N17.x, N18.x, N19.x, Q61.xx, I12, I13, N18, N19, N29, Z99.2, Z49, Q61, O10.2, O10.3 |
| Hypertension (HTN) | 401.xx,402.xx,403.xx,404.xx,405.xx | I10, I11.0, I11.9, I12.0, I12.9, I13.0, I13.2, I13.11, I15.xx, N26.2 |
| Chronic obstructive lung disease (COPD) | 491.xx,492.xx, 494.xx, 495.xx, 496.xx | J41.x, J42.x, J43.x, J44.x, J47.x, J67.x |
| **Drug** | **ATC code** | |
| Aspirin | B01AC06 | |
| ACEI | C09AA; C09B, ARB: C09CA; C09D | |
| Anti-coagulants: Warfarin | B01AA03; B01AE07; B01AF01; B01AF02; B01AF03 | |
| ARB | C09CA; C09D | |
| Statin | C10AA01- C10AA09 | |
| Cyproterone, bicalutamid and diethylstilbestrol | G03HA01; L02BB03; L02AA01 | |
